# The Role of Next-Generation Sequencing in Cardiovascular Disease: A New Era of Precision Cardiology

**DOI:** 10.3390/life16050796

**Published:** 2026-05-10

**Authors:** Konstantinos Agiannitopoulos, Anastasios Papageorgiou, Elisavet Kouvidi, Eleni Kalampoka, Anna Papadopoulou, Anastassios Philippou, Stavroula Papadodima, Lubomir L. Traikov, Dimitrios C. Angouras

**Affiliations:** 1 Division of Genetics & Biotechnology, Department of Biology, National and Kapodistrian University of Athens, 15772 Athens, Greece; 2 Department of Physiology, Medical School, National and Kapodistrian University of Athens, 11527 Athens, Greece; tasos1998p@hotmail.com (A.P.); tfilipou@med.uoa.gr (A.P.); 3 Department of Medical Physics and Biophysics, Medical University, 1431 Sofia, Bulgaria; lltraikov@medfac.mu-sofia.bg; 4 Department of Genetics, Institute of Child Health, 11527 Athens, Greece; ekouvidi@gmail.com; 5 Laboratory of Clinical Biochemistry, Medical School, University General Hospital ‘Attikon’, National and Kapodistrian University of Athens, 12462 Athens, Greece; elenikalamp@yahoo.gr (E.K.); anpapado@med.uoa.gr (A.P.); 6 Department of Forensic Medicine and Toxicology, Medical School, National and Kapodistrian University of Athens, 11527 Athens, Greece; stpapd@med.uoa.gr; 7 Department of Cardiac Surgery, Attikon University Hospital, Medical School, National and Kapodistrian University of Athens, 11527 Athens, Greece; dangouras@yahoo.com

**Keywords:** next-generation sequencing, cardiovascular disease, precision medicine, polygenic risk score, pharmacogenomics

## Abstract

Cardiovascular diseases (CVDs) are the foremost contributor to global mortality, with a significant inherited factor that has long been recognized but only recently become decipherable. Next-generation sequencing (NGS) has transformed the study of cardiovascular genetics, allowing researchers to move beyond single-gene analyses toward comprehensive assessments of both rare and common genetic variations. This review summarizes how NGS informs clinical practice, from the molecular diagnosis of inherited cardiac disorders and risk prediction using polygenic models to emerging applications in precision therapeutics. It also discusses analytical and ethical challenges and highlights new technologies, such as long-read and single-cell sequencing, that are likely to further advance precision cardiology.

## 1. Introduction

Cardiovascular diseases (CVDs), encompassing ischemic heart disease, stroke, and various arrhythmias, continue to be the leading cause of death worldwide, responsible for nearly 18 million deaths each year. Both inherited and acquired factors contribute to the risk of developing these conditions [1,2]. Beyond mortality, CVDs impose a significant burden on healthcare systems and society, accounting for substantial years of life lost and disability-adjusted life years, despite advances in prevention, diagnosis, and treatment [3,4].

Genetic contribution to cardiovascular conditions includes rare, high-impact single-gene pathogenic variations causing monogenic disorders, such as hypertrophic cardiomyopathy and familial hypercholesterolemia, and more frequently common genetic variants, each exerting a modest influence on overall risk associated with complex, polygenic cardiovascular diseases like atrial fibrillation (AF) and coronary artery disease (CAD) [5,6,7]. The later, genetic susceptibility is further modulated by environmental, lifestyle, and metabolic factors, including diet, smoking, blood pressure, and diabetes. Understanding this genetic architecture, from rare, high-impact mutations to common, low-effect variants, is essential for uncovering disease mechanisms and advancing precision cardiology [8].

The first disease-causing gene identified for a monogenic cardiovascular disorder was *MYH7*, encoding the β-myosin heavy chain, in familial hypertrophic cardiomyopathy (HCM). In 1989, linkage analysis of large multigenerational families with autosomal dominant HCM mapped the disease locus to chromosome 14q11–q12 using polymorphic DNA markers. A candidate gene approach subsequently identified *MYH7* as a biologically plausible gene within this region, and Sanger sequencing in 1990 revealed missense mutations that co-segregated with disease and were absent in unaffected individuals [9]. Initial studies on the discovery of the genetic basis of CVDs relied on laborious and time-consuming methodologies like linkage analysis and Sanger sequencing to trace and identify heritable markers in families [10].

The completion of the Human Genome Project in 2003 provided the foundational blueprint that revolutionized the study of genetic disorders. In cardiology, this enabled a systematic shift from linkage analysis and candidate gene approaches to the comprehensive characterization of variants underlying inherited cardiac conditions. Next-generation sequencing (NGS) has been instrumental in this paradigm shift, allowing for the simultaneous interrogation of numerous genes and illuminating the molecular etiology of diseases once categorized solely by clinical phenotype [11]. Over the past twenty years, declining sequencing costs have broadened access to these technologies, facilitating genome-wide investigations and the incorporation of NGS into routine clinical practice [12]. These advances have elucidated the complex genetic architecture of CVDs and enhanced the practicality of comprehensive clinical genetic testing [13].

This review aims to provide a comprehensive overview of the profound impact of NGS on the entire spectrum of cardiovascular medicine. NGS technological principles, including comparison of whole-genome, whole-exome, and targeted panel approaches, are described. In addition, its clinical applications in diagnosis, risk stratification, and therapy selection are addressed. Analytical and ethical considerations, including variant interpretation and incidental findings, are also discussed. Finally, emerging technologies, such as long-read sequencing, single-cell genomics, and artificial intelligence, are highlighted for their potential to strengthen the role of genomics in precision cardiovascular care.

## 2. The NGS Pipeline: From DNA to Data

DNA sequencing allows the “reading” and, subsequently, the analysis and interpretation of the information hidden in the precisely ordered nucleotide sequence of the DNA of any origin. Since its first introduction in the 1970s, sequencing has improved mainly due to the rapid advancement in technology and bioinformatics. Next-generation sequencing is the evolution of direct first-generation sequencing that enables the parallel analysis of millions of DNA fragments, allowing broad genomic assessment in a single run. Today, we are able to study part of our genome, only some genes, the whole exome (WES) or our whole genome (WGS). The constantly improved platforms and the corresponding data analysis software dramatically reduce the laboratory work time, as well as the cost of the analysis. The choice of the platform depends on the needs of the test, for instance, the depth, coverage, etc. [14].

**Figure 1:**
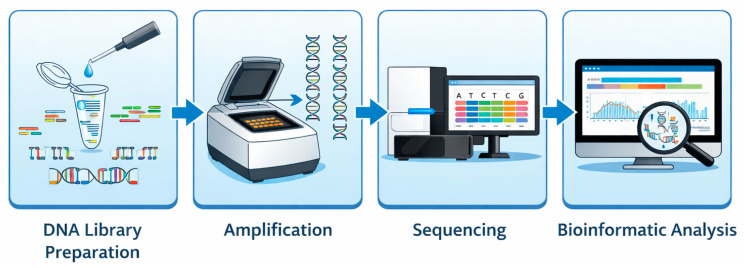
The four major steps of next-generation sequencing (NGS).

Despite platform differences, most methodologies include four essential laboratory stages: DNA library preparation, amplification, sequencing, and bioinformatic analysis, each influencing data quality and clinical interpretation (Figure 1) [14].

During library preparation, DNA is fragmented and short DNA sequences, called adapters, are added for capturing and indexing specific regions of the testing samples. The amplification of the captured regions based on different protocols increases the signal strength, and sequencing proceeds nucleotide by nucleotide, with detection based on fluorescent imaging or pH changes [15,16,17,18,19]. By the end of each run, billions of short sequences (short reads) are generated and the so-called raw data are collected. Long-read sequencing complements NGS by resolving complex genomic regions and structural variants beyond the limits of short reads [15,16,17,18,19].

Bioinformatic analysis, essential to transform the collected raw signals into interpretable results, consists of three levels of data analysis: the primary analysis that converts optical or voltage signals obtained from sequencing reactions into base calls (reads) with respective quality scores (probability of base-calling errors are essential for assessing read reliability and downstream variant accuracy), and the secondary analysis that aligns the reads to a reference genome and identifies variants, including single-nucleotide variations (SNVs), small insertions/deletions (indels), and structural changes. Finally, the tertiary analysis interprets variants using genomic annotations, clinical data, and frameworks such as the American College of Medical Genetics and Genomics (ACMG) guidelines [20].

The ACMG guidelines provide a standardized framework for the interpretation and classification of genetic variants to ensure consistency and clinical validity in genomic testing. Variants are classified into five categories—pathogenic, likely pathogenic, variant of uncertain significance (VUS), likely benign, and benign—based on weighted evidence types, including population frequency, computational predictions, functional studies, segregation data, de novo occurrence, and previous reports in affected individuals. Evidence is graded by strength (very strong, strong, moderate, supporting) and combined using defined rules to reach a final classification. These guidelines are widely used in clinical genetics to support accurate diagnosis, reporting, and patient management across inherited diseases [21].

As the NGS pipeline is a multistep process, generating thousands of billions of signals that are transformed into readable information through bioinformatics, each step requires a certain level of quality (quality score) to be acceptable. Because each step depends on the previous one, errors can propagate throughout the pipeline, affecting clinical interpretations [21,22]. For instance, low-quality raw data may result in inaccurate base calls in primary analysis, and thus to false variants and wrong interpretations [23], while misalignments in secondary analysis, especially in regions with pseudogenes, may misattribute benign variants to disease genes [24]. Finally, the interpretation of all information collected during the previous steps must follow commonly accepted rules and use updated databases to avoid variant misclassification and deliver safe and effective clinical genetic reports [25,26,27].

**Figure 2:**
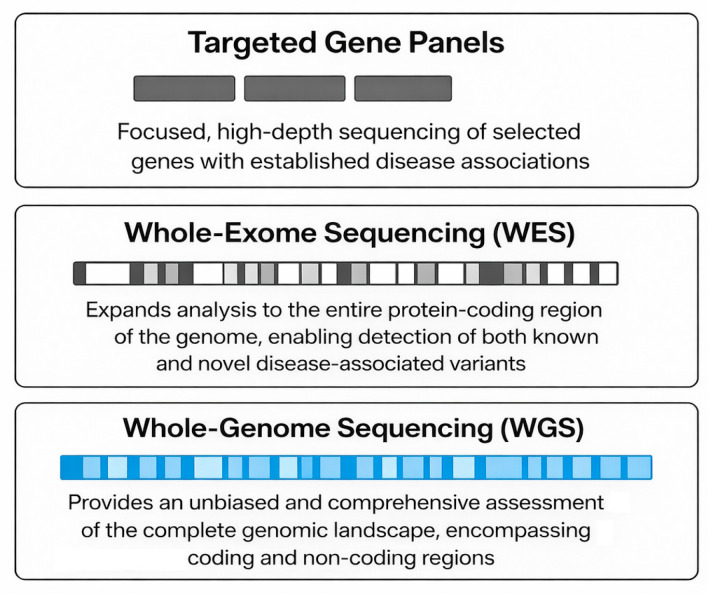
Schematic representation of the hierarchical spectrum of NGS approaches.

Three principal next-generation sequencing (NGS) approaches are commonly applied: targeted gene panels, whole-exome sequencing (WES), and whole-genome sequencing (WGS), each providing a distinct balance of scope, cost, and interpretive complexity (Table 1, Figure 2) [28].

Targeted gene panels have clinical applications. They include a predefined set of well-characterized genes, associated with genetic diseases. This selection offers a higher sequencing depth, rapid turnaround, and cost-effectiveness. By focusing on genes with well-established disease associations, these panels achieve high diagnostic accuracy while minimizing incidental or secondary findings [29,30]. When studying patients with atypical or overlapping phenotypes that do not clearly conform to a single diagnostic category, WES expands the analysis to all coding regions of the genome, capturing the majority of known pathogenic variants. WES is especially valuable in patients. However, this broader approach increases the number of VUSs and demands more advanced analytical pipelines and interpretation [31,32].

WGS provides the most comprehensive view, encompassing non-coding regions, regulatory elements, and structural variants. While WGS offers the highest potential for novel variant discovery, it also entails greater financial and computational requirements. As analytical tools and reference databases continue to improve, WGS is expected to evolve from a primarily research-based technique into a core component of clinical cardiogenomics [33,34,35].

In clinical practice, the selection among these NGS strategies depends on the specific scenario, available resources, and diagnostic goals. Targeted panels are preferred for straightforward cases requiring efficiency and clarity, WES for unexplained or complex phenotypes, and WGS when maximal discovery potential is desired. Collectively, these approaches constitute a hierarchical continuum, enabling personalized genetic diagnosis and management across the spectrum of inherited and complex cardiovascular diseases.

## 3. Clinical Applications of NGS in Cardiology

**Figure 3:**
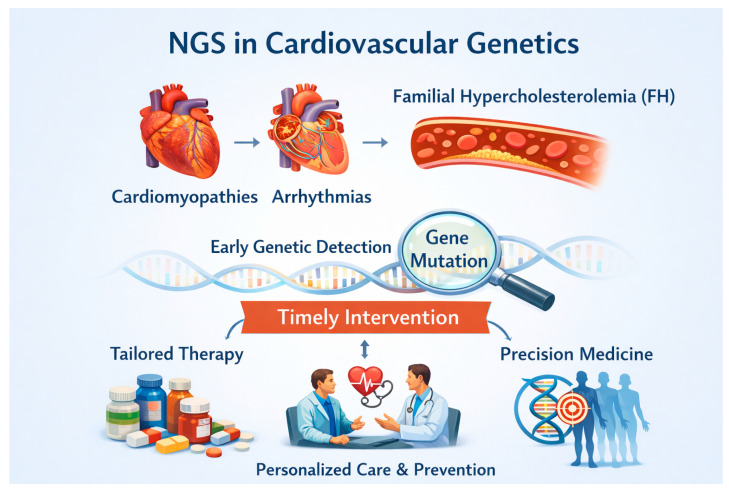
Schematic representation of clinical applications of NGS for different cardiovascular diseases.

The adoption of NGS has fundamentally transformed the application of genetics in cardiovascular care [36]. The most prominent diseases that can be characterized by NGS include those that follow Mendelian penetrance including cardiomyopathies, arrhythmogenic diseases affecting the electric conduit electrical conduction system of the heart but also diseases affecting the vascular part such as familial hypercholesterolemia (FH). The value of the early detection of genes and mutations related to the above disorders is crucial because it enables timely intervention to prevent or delay phenotypic expression. Management strategies differ for each disease and each patient, consistent with personalized precision medicine (Figure 3) [37].

Generally, management of these disorders includes medical and mechanical interventions through understanding the genetic and molecular basis of them by the use of NGS. Pharmacologic approaches such as antiarrhythmics blocking sodium and potassium channels, beta-blockers, calcium channel blockers as well as antihypertensive medications, therapies for idiopathic pulmonary hypertension, and lipid-lowering agents are extremely important for the early prevention of morbidity and mortality of these patients. In addition, the early detection of mutations of genes associated with disturbances of the electrical conduction system has improved the involvement of mechanical applications such as the installment of pacemakers and defibrillators that prolong life expectancy. Finally, one recently developed area related to NGS approaches in cardiology involves the application of it into mutations associated with genes of mitochondrial DNA [38]. Mitochondrial DNA mutations are associated with an increased risk of atherosclerosis, coronary heart disease, cardiomyopathies as well as the formation of reactive oxygen species that are extremely toxic for cardiac muscle and contractility [39].

NGS can clarify ambiguous cardiovascular phenotypes and confirm clinical suspicions. For instance, the detection of a pathogenic variant in a patient with borderline left ventricular hypertrophy can distinguish sarcomeric hypertrophic cardiomyopathy from secondary hypertrophy caused by hypertension or athletic remodeling. Similarly, in individuals presenting with unexplained arrhythmias, sequencing can uncover concealed channelopathies or cardiomyopathies, converting uncertain clinical findings into precise genetic diagnoses [40].

When a disease-causing variant is identified, testing relatives can facilitate early detection and proactive management within families. Family screening of mutation carriers enables the identification of at-risk individuals prior to the onset of clinical symptoms, supporting surveillance programs, lifestyle interventions, and timely initiation of therapies to prevent adverse cardiac events [41].

**Figure 4:**
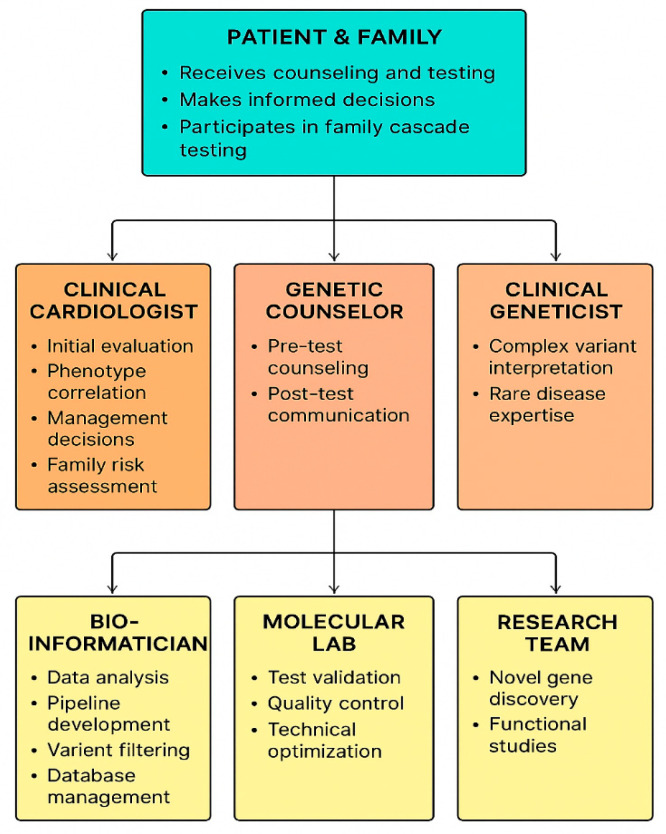
End-to-end workflow of clinical NGS in cardiology. Schematic overview of the integrated clinical and analytical NGS pipeline.

In inherited cardiomyopathies, such as those associated with *LMNA* (lamin A/C) gene mutations, the genotype can guide decisions regarding primary prevention implantable cardioverter defibrillator placement, even in asymptomatic individuals with preserved ventricular function [42]. Likewise, genotype-driven management in conditions like long QT syndrome or catecholaminergic polymorphic ventricular tachycardia helps tailor drug therapy, exercise recommendations, and lifestyle counseling [42]. These examples illustrate the transition of molecular information from a purely descriptive tool to a critical determinant in clinical decision-making (Figure 4).

## 4. Molecular Diagnosis of Mendelian Cardiovascular Diseases

Mendelian cardiovascular diseases, characterized by their inheritance in predictable patterns (e.g., autosomal dominant, recessive) and often high penetrance, are prime targets for NGS-based diagnosis. These disorders primarily include inherited cardiomyopathies, channelopathies, and familial metabolic conditions such as familial hypercholesterolemia. The genetic architecture ranges from single-nucleotide variants (SNVs) in sarcomeric genes to larger structural variations affecting cytoskeletal or ion channel components. Furthermore, the expression of these monogenic disorders can be influenced by genetic modifiers, environmental factors, and complex mechanisms, such as alternative splicing and post-translational modifications, which contribute to variable penetrance and expressivity observed within affected families [43,44].

NGS-based multi-gene panels have consequently emerged as the standard of care for the genetic evaluation of most inherited cardiac conditions. This approach enables the simultaneous interrogation of numerous genes with established or emerging disease associations, offering markedly improved diagnostic yield and efficiency compared with traditional sequential single-gene testing [45]. Identification of a pathogenic or likely pathogenic variant provides a definitive molecular diagnosis, confirming or refining the clinical diagnosis and clarifying the underlying genetic etiology. Importantly, establishing a molecular diagnosis facilitates the targeted cascade screening of asymptomatic at-risk relatives, enabling the early identification, surveillance, and implementation of preventive or risk-reducing interventions. Furthermore, in selected contexts, genotype information contributes to risk stratification, prognostic assessment, and personalized therapeutic decision-making, for example, by influencing pharmacological management or the consideration of implantable cardioverter defibrillator (ICD) therapy [46,47].

### 4.1. Genetic Basis and Molecular Diagnosis of Inherited Cardiomyopathies

#### 4.1.1. Hypertrophic Cardiomyopathy

Hypertrophic cardiomyopathy (HCM) is the most prevalent inherited cardiac disease, affecting approximately one in 500 individuals worldwide [48]. It is characterized by unexplained left ventricular hypertrophy in the absence of secondary causes such as hypertension or valvular disease [48]. The disorder follows an autosomal dominant inheritance and primarily arises from pathogenic variants in sarcomeric genes, notably *MYBPC3 (encoding myosin-binding protein C)* and *MYH7 (encoding β-myosin heavy chain)*, which encode sarcomeric proteins essential for cardiac muscle contraction [49]. Mutations in these genes together account for roughly 30–60% of genetically confirmed adult cases [50]. These variants impair sarcomere organization and contractile efficiency, resulting in cardiomyocyte hypertrophy, myofibrillar disarray, and interstitial fibrosis. Truncating *MYBPC3* mutations typically manifest later and produce a milder phenotype, whereas missense *MYH7* mutations often cause earlier onset and more pronounced hypertrophy [51,52].

#### 4.1.2. Dilated Cardiomyopathy

Dilated cardiomyopathy (DCM) is a myocardial disorder with diverse genetic and clinical presentations, characterized by left ventricular enlargement and reduced systolic function in the absence of abnormal loading conditions or coronary artery disease [53]. Familial DCM is predominantly inherited in an autosomal dominant pattern and the most common genetic contributors are truncating variants in the *TTN* (*Titin*) gene, encoding the giant sarcomeric protein titin, present in approximately 15–25% of cases. These mutations compromise sarcomere integrity and myocardial contractility [54]. Pathogenic variants in the *LMNA* (*Lamin A/C*) gene, encoding lamin A/C of the nuclear envelope, are also clinically significant. *LMNA-*related DCM is often associated with early conduction system disease and life-threatening ventricular arrhythmias, sometimes preceding overt left ventricular dysfunction [55]. Early identification of *LMNA* mutations is essential for prognostic assessment, guiding enhanced monitoring and timely consideration of prophylactic implantable cardioverter defibrillator (ICD) therapy. Additional genes implicated in DCM include *DSP*, *FLNC*, and *RBM20*, highlighting the multifactorial molecular etiology of the disease [56].

#### 4.1.3. Arrhythmogenic Right Ventricular Cardiomyopathy

Arrhythmogenic right ventricular cardiomyopathy (ARVC) is a genetically mediated myocardial disorder characterized by fibro-fatty replacement of the right ventricular myocardium, predisposing to ventricular arrhythmias and sudden cardiac death, particularly in young individuals and athletes [54]. It is caused predominantly by mutations in desmosomal genes, including *PKP2* (*plakophilin-2*), *DSP* (*desmoplakin*), *DSG2* (*desmoglein*-2), and *DSC2* (*desmocollin-2*), which are responsible for mechanical adhesion between cardiomyocytes [57]. Variants in *PKP2* are the most frequent, identified in approximately 50–60% of genotype-positive cases [58]. Impaired desmosomal integrity leads to myocyte detachment and apoptosis under mechanical stress, culminating in fibro-fatty replacement of the right ventricular myocardium and an elevated risk of arrhythmias [59]. ARVC is typically inherited in an autosomal dominant pattern with incomplete penetrance, but environmental factors such as intense endurance exercise can accelerate disease onset and progression [60,61].

Cooperatively, the major inherited cardiomyopathies underscore the pivotal importance of molecular genetics in illuminating disease pathways, enhancing clinical diagnosis, and tailoring precision management (Table 2). Although these disorders display a distinct morphological signature, such as the left ventricular thickening seen in hypertrophic cardiomyopathy (HCM), ventricular dilation characteristic of dilated cardiomyopathy (DCM), or the fibro-fatty myocardial substitution typical of arrhythmogenic cardiomyopathy (ACM), they all stem from the disruption of core structural or functional elements within cardiomyocytes, including the sarcomere, cytoskeleton, nuclear envelope, and intercellular junctions. The fact that genes such as *DSP*, *FLNC* and *LMNA* are implicated across more than one cardiomyopathy phenotype highlights the overlapping genetic architecture and the continuum that connects these entities [62]. This pleiotropy means that overlapping or evolving phenotypes may appear within the same individual or family, emphasizing the need for broad gene panel testing and periodic clinical reevaluation. By integrating genetic data with in-depth phenotypic assessment, clinicians can sharpen diagnostic accuracy, refine prognostic estimates, and support familial risk stratification, all key components of contemporary precision cardiology.

Although multi-gene targeted panels remain the workhorse for genetic diagnosis, newer genomic strategies are broadening the horizon of inherited cardiomyopathy research and clinical applications. WES and WGS permit the detection of variants beyond classical gene sets, including those in regulatory domains, deep intronic regions, and novel loci not captured by standard panels [63]. These high-throughput approaches have revealed additional pathogenic mechanisms such as splicing alterations, copy number changes and complex structural rearrangements.

Emerging evidence also indicates that disease manifestation is not simply determined by individual pathogenic variants; rather, the broader genetic background—including modifier genes and polygenic risk factors—modulates penetrance and phenotype severity. Combining polygenic risk scores (PRSs) with monogenic findings thus holds promise for a more refined individualized risk stratification and prognostic evaluation. Moreover, the advent of multi-omics methodologies—spanning transcriptomics, proteomics and metabolomics—together with induced pluripotent stem cell (iPSC) models is advancing our understanding of genotype–phenotype relationships and opening pathways toward targeted therapies [64]. As these technologies mature, the future of cardiomyopathy genomics is likely to emphasize integrated, data-driven frameworks that link molecular discovery to clinical translation, thereby elevating precision in diagnosis, treatment and prevention.

Despite considerable progress in genomic technology and variant interpretation, several obstacles hinder the full realization of precision medicine in inherited cardiomyopathies. These include the frequent occurrence of variants of uncertain significance (VUSs), incomplete or age-related penetrance, and substantial phenotypic heterogeneity, particularly in sporadic or borderline cases. In addition, underrepresentation of diverse ancestries in genetic reference databases limits the accuracy of variant classification and equitable access to genomic insights. Global collaborative endeavors to enlarge population diversity, standardize variant curation and integrate longitudinal genotype–phenotype data are therefore essential. Ultimately, by synergizing high-resolution genomics with clinical, imaging and biomarker datasets, the field aims to refine disease classification, improve risk stratification and enable genuinely individualized management strategies—advancing toward the goal of truly personalized cardiovascular care [65].

### 4.2. Genetic Basis and Molecular Diagnosis of Inherited Arrhythmia Syndromes

#### 4.2.1. Long QT Syndrome

Long QT syndrome (LQTS) is a genetic cardiac channelopathy characterized by delayed ventricular repolarization, which predisposes affected individuals to potentially fatal arrhythmias. NGS panels have become a pivotal diagnostic tool, identifying pathogenic variants in more than 75% of patients with a definitive clinical diagnosis and highlighting strong genotype–phenotype relationships [66].

The majority of LQTS cases are linked to mutations in a small number of key ion channel genes. The *KCNQ1* (*Potassium Voltage-Gated Channel Subfamily Q Member 1*) (LQT1) gene encodes the α-subunit of the slow delayed rectifier potassium current (IKs); pathogenic variants reduce potassium outflow, prolonging the QT interval. Patients with *KCNQ1* mutations are especially vulnerable to arrhythmias triggered by exercise, such as swimming or sudden exertion. The *KCNH2* (*Potassium Voltage-Gated Channel Subfamily H Member 2*) (LQT2) gene encodes the α-subunit of the rapid delayed rectifier potassium current (IKr); mutations often lead to arrhythmic events provoked by auditory stimuli or acute emotional stress. The *SCN5A* (*Sodium Voltage-Gated Channel Alpha Subunit 5*) (LQT3) gene encodes the cardiac sodium channel Nav1.5, and mutations typically cause a persistent inward sodium current during repolarization, increasing arrhythmic risk at rest or during sleep [67].

Knowledge of the genotype is crucial for risk stratification and therapeutic decision-making. β-blockers are highly effective in LQT1 and LQT2, whereas sodium channel blockers, such as mexiletine, may benefit LQT3 patients. Lifestyle measures, including avoidance of QT-prolonging medications, stress management, and individualized exercise restrictions, are also guided by genotype [67].

#### 4.2.2. Brugada Syndrome

Brugada Syndrome (BrS) is an inherited arrhythmogenic disorder defined by ST segment elevation in the right precordial leads and an increased risk of sudden cardiac death from ventricular fibrillation. The condition follows an autosomal dominant inheritance pattern with variable penetrance, meaning not all mutation carriers exhibit clinical or electrocardiographic (ECG) manifestations. Pathogenic variants in the *SCN5A* gene, encoding the α-subunit of the cardiac sodium channel Nav1.5, represent the most frequent genetic cause. These mutations reduce the sodium current, causing conduction delays, particularly in the right ventricular outflow tract. The overall diagnostic yield is approximately 20%, reflecting substantial genetic heterogeneity, polygenic influences, and complex inheritance patterns. Rare variants in other genes have also been implicated [68].

Arrhythmic events can be precipitated by fever, certain medications, or electrolyte disturbances. Genetic testing confirms the diagnosis in some patients and supports family screening, although a negative result does not exclude the syndrome. Risk stratification primarily depends on clinical features such as a prior syncope, spontaneous type 1 ECG pattern, and family history of sudden cardiac death. Management may include implantable cardioverter defibrillators (ICDs), avoidance of known triggers, and, in select cases, pharmacologic therapy such as quinidine [68].

#### 4.2.3. Catecholaminergic Polymorphic Ventricular Tachycardia

Catecholaminergic polymorphic ventricular tachycardia (CPVT) is a rare, inherited arrhythmia syndrome characterized by exercise- or stress-induced polymorphic ventricular tachycardia in individuals with structurally normal hearts and often normal resting ECGs. Untreated CPVT carries a high risk of sudden cardiac death during physical exertion or emotional stress [69].

The most commonly affected gene is *RYR2 (Ryanodine Receptor 2*), which encodes the cardiac ryanodine receptor responsible for calcium release from the sarcoplasmic reticulum. Pathogenic variants are detected in approximately 60% of cases. Less common autosomal recessive forms involve the *CASQ2* (*Calsequestrin 2*) gene, encoding calsequestrin 2, a calcium-binding protein. Mutations in these genes disrupt intracellular calcium handling, triggering delays after depolarizations and arrhythmias under catecholaminergic stimulation. Because resting ECGs may appear normal, genetic testing is critical, especially for patients with a suggestive clinical or family history. Identification of pathogenic variants guides early interventions, including β-blocker therapy, avoidance of strenuous activity, and ICD implantation for high-risk individuals. Cascade screening allows the identification of asymptomatic relatives and preventive care [69].

NGS-based genetic testing is a cornerstone for evaluating inherited arrhythmias. It provides a high diagnostic yield in LQTS, confirms a subset of BrS cases while highlighting genetic complexity, and is essential for accurate CPVT diagnosis when clinical signs are subtle [70] (Table 3).

### 4.3. Genetic Basis and Molecular Diagnosis of Familial Hypercholesterolemia

Familial hypercholesterolemia (FH) is a common inherited disorder affecting lipid metabolism, marked by the lifelong elevation of low-density lipoprotein cholesterol (LDL-C), which significantly elevates the risk of premature atherosclerotic cardiovascular disease (ASCVD), if left untreated. FH is most often inherited in an autosomal dominant manner. In the rarer homozygous or compound heterozygous forms, the phenotype is far more severe, often leading to cardiovascular events in childhood or early adulthood [71].

FH is most frequently linked to pathogenic variants in three primary genes, *LDLR* (*Low-Density Lipoprotein Receptor*), *APOB* (*Apolipoprotein B*) and *PCSK9* (*Proprotein Convertase Subtilisin/Kexin Type 9*), each disrupting LDL-C metabolism by different mechanisms. LDLR variants impair the function of hepatic LDL receptors, thereby decreasing the clearance of LDL particles and producing markedly high LDL-C levels, especially in homozygotes. *APOB* variants interfere with the binding of LDL particles to hepatic receptors, reducing LDL uptake, resulting in elevated circulating LDL-C; the phenotype is typically milder than for null *LDLR* mutations but still substantially increases ASCVD risk. *PCSK9 gain*-of-function variants lead to accelerated receptor degradation, further raising LDL-C levels. Though less common than *LDLR* or *APOB* defects, *PCSK9* mutations can generate very high LDL-C, especially when combined with other genetic or environmental risks [71].

Together, these three genes account for the majority of genetically defined FH cases. NGS panels are increasingly central in FH diagnosis, detecting pathogenic variants in approximately 40–60% of individuals who meet the clinical criteria for definite FH [72]. Genetic confirmation offers multiple benefits: firstly, it provides diagnostic clarity in atypical or borderline presentations where standard criteria may be inconclusive. Secondly, it enables the cascade genetic screening of first-degree relatives, critical in an autosomal dominant condition where each child of an affected parent has a 50% chance of inheriting the variant. Early identification of asymptomatic carriers allows pre-emptive interventions, lifestyle modifications, and pharmacotherapy, to reduce the risk of premature ASCVD [73]. Thirdly, genetic results assist in prognostic assessment; for example, *LDLR* null-alleles or biallelic mutations are associated with markedly higher LDL-C levels, earlier onset of cardiovascular events, and sometimes the need for more intensive treatment [74].

## 5. Risk Stratification for Complex Cardiovascular Diseases

Coronary artery disease (CAD) and atrial fibrillation (AF) are among the most common complex cardiovascular diseases. CAD is characterized by the progressive accumulation of atherosclerotic plaques within the coronary arteries, leading to myocardial ischemia and infarction, and is driven by a complex interplay of metabolic, inflammatory, and environmental factors [75]. In contrast, AF is the most common sustained cardiac arrhythmia, defined by disorganized atrial electrical activity and ineffective atrial contraction, and is associated with an increased risk of stroke, heart failure, and mortality [76]. Although distinct in their pathophysiology, both conditions share overlapping risk factors, including aging, hypertension, obesity, and diabetes, and are increasingly recognized as complex diseases with substantial genetic contributions. Understanding their genetic architecture is therefore critical for improving risk stratification, prevention, and therapeutic strategies [75,76].

Recent advances in NGS technologies have substantially reshaped our understanding of the genetic basis of complex cardiovascular diseases, particularly CAD and AF. Early genetic models were largely grounded in a monogenic paradigm, in which single, high-penetrance mutations were considered sufficient to cause disease. While this framework remains highly relevant for rare inherited cardiovascular disorders, it does not adequately explain the genetic architecture of common conditions such as CAD and AF, which arise from a far more intricate interplay of genetic and environmental factors [77].

Accumulating evidence now supports a polygenic model of disease susceptibility, whereby risk is determined by the combined effect of numerous genetic variants, each contributing modestly to overall disease predisposition. This conceptual shift has been driven largely by genome-wide association studies (GWASs), which enable the systematic interrogation of genetic variation across large populations [78]. Through these studies, hundreds of loci associated with CAD and AF have been identified, the majority of which reside in non-coding regions of the genome and are believed to regulate gene expression rather than directly altering the protein structure.

In CAD, GWAS findings have consistently implicated pathways related to lipid metabolism, vascular inflammation, endothelial dysfunction, and plaque stability [79]. Variants influencing LDL cholesterol, particularly those near genes such as *LDLR*, *APOB*, and *PCSK9*, have provided strong genetic validation of lipid-driven atherogenesis as a central mechanism of disease [80]. In parallel, loci such as chromosome 9p21 highlight pathways that appear to operate independently of traditional risk factors, underscoring the complexity of atherosclerotic disease biology [81]. More recent large-scale studies have further extended these findings by demonstrating that polygenic models incorporating millions of variants can meaningfully stratify individuals according to lifetime risk. Individuals in the highest percentiles of polygenic risk may exhibit a several-fold increase in CAD risk, in some cases approaching that observed in monogenic disorders [82].

The genetic architecture of atrial fibrillation reflects its distinct electrophysiological and structural underpinnings. GWASs have identified key loci associated with cardiac development, ion channel function, and atrial remodeling. Among these, variants near PITX2 represent some of the most robust genetic signals linked to AF, highlighting the importance of developmental pathways in arrhythmogenesis [83]. Emerging evidence suggests that the interaction between genetic susceptibility and acquired risk factors such as hypertension, obesity, and aging plays a critical role in determining AF onset, progression, and recurrence [84].

Polygenic risk scores (PRSs) provide a quantitative summary of inherited susceptibility by aggregating information from many genetic variants identified in GWASs. Early PRSs were based on a limited number of strongly associated variants, whereas contemporary methods incorporate millions of single-nucleotide polymorphisms (SNPs) across the genome, applying advanced statistical models that account for correlations among nearby variants to improve accuracy. Despite these advancements, a major challenge is population diversity: most GWASs have been conducted in individuals of European ancestry, and PRSs derived from these datasets often perform suboptimal when applied to African, Asian, or Hispanic populations. Addressing this limitation is a key focus of current research aiming to build equitable, generalizable risk models [85].

Clinically, PRSs allow the identification of high-risk individuals long before disease manifests. For example, individuals with elevated CAD-related PRSs can experience a two-to-fourfold greater lifetime risk, comparable to the risk observed in certain monogenic conditions such as familial hypercholesterolemia. This underscores that the cumulative burden of common variants can have effects as significant as rare, single-gene mutations [86].

The greatest utility of PRSs is realized when combined with traditional risk factors such as age, sex, blood pressure, cholesterol, smoking, and diabetes. Integration of genomic and clinical data refines risk prediction, enabling the reclassification of individuals who may be misestimated by conventional models. For instance, a patient with an intermediate clinical risk but a high PRS might benefit from the earlier initiation of preventive therapies such as statins and targeted lifestyle interventions. Incorporating PRSs into clinical workflows allows personalized preventive strategies, improving the precision of cardiovascular care [87].

**Figure 5:**
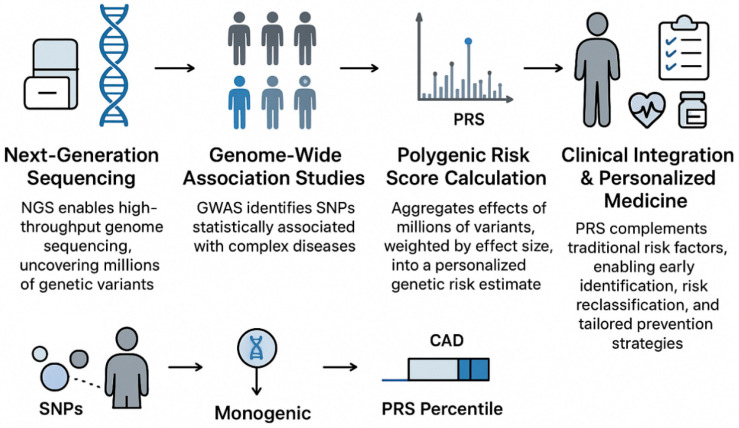
From genetic discovery to personalized risk prediction. The workflow from large-scale genetic discovery to individualized risk assessment using polygenic risk scores (PRSs).

In summary, polygenic risk scores serve as a critical link between large-scale genetic discoveries and individualized patient management. By capturing the diffuse genetic contribution to disease risk, PRSs complement traditional risk assessments, support proactive prevention, and have the potential to become a cornerstone of precision cardiovascular medicine [88] (Figure 5).

NGS technologies have also been applied to other cardiovascular diseases with complex traits, such as aortic aneurysms. Aortic aneurysms and genetic syndromes associated with them including Marfan, Ehlers–Danlos and Loeys–Diets syndrome are connected to mutations causing disturbances in the normal elasticity durability of the aorta [89]. A lot of children as well as adolescents and adults have phenotypes similar or completely different for each syndrome based on the extent, type and area of mutation resulting in functional, partial dysfunctional and completely non-functional proteins associated with the respective involved genes [90]. Studies demonstrated the feasibility of NGS for detecting rare variants in large cohorts of aortic aneurysm patients [91]. Subsequent investigations using WES and targeted gene panels have further highlighted the diagnostic utility of NGS in thoracic aortic aneurysms and dissections (TAADs), identifying both known and novel pathogenic variants in disease-associated genes [92].

Moreover, NGS has enabled the discovery of causal mutations in genes such as *FBN1*, contributing to familial forms of aneurysm and improving our understanding of genotype–phenotype correlations [93]. Beyond variant detection, transcriptomic NGS approaches have also revealed dysregulated pathways, for instance, DNA repair and oxidative stress mechanisms, in abdominal aortic aneurysms, offering potential biomarkers and therapeutic targets [94]. Taken together, these studies underscore the significant potential of NGS not only for identifying genetic susceptibility in aortic aneurysms but also for advancing precision medicine through improved risk stratification, early diagnosis, and the development of targeted therapies.

## 6. Guiding Personalized Therapy (Pharmacogenomics)

NGS has revolutionized pharmacogenomics by enabling the rapid and comprehensive identification of genetic variants that influence drug response. This genomic information allows clinicians to personalize therapy, enhancing drug efficacy while reducing the likelihood of adverse reactions. Several clinically significant examples demonstrate how NGS-guided pharmacogenetics can optimize treatment and improve patient safety [95].

Warfarin, a widely used anticoagulant with a narrow therapeutic index, exemplifies the importance of pharmacogenomic guidance. Genetic variants in the *VKORC1* (*Vitamin K epoxide reductase complex 1*) and *CYP2C9* (*cytochrome P450 2C9*) genes can significantly influence warfarin metabolism, determining whether patients require higher or lower doses to achieve effective anticoagulation. Recognizing these genetic differences, the FDA now incorporates pharmacogenetic dosing recommendations into the warfarin label, supporting the genotype-informed initiation of therapy and helping reduce the risk of bleeding or thrombotic complications [96].

Clopidogrel, an antiplatelet drug commonly prescribed following coronary stent placement, provides another example of genotype-guided therapy. Its activation depends on the enzyme encoded by the *CYP2C19* (*cytochrome P450 2C19*) gene, and individuals with loss-of-function alleles are classified as poor metabolizers. Such patients exhibit reduced conversion to clopidogrel’s active form, leading to diminished platelet inhibition and a higher risk of thrombotic events, including stent thrombosis. Clinical guidelines advise considering alternative agents, such as prasugrel or ticagrelor, for poor metabolizers, particularly after percutaneous coronary intervention, to ensure adequate antiplatelet protection [97].

Statin-induced myopathy illustrates pharmacogenetic guidance in lipid-lowering therapy. Variants in the *SLCO1B1* (*Solute Carrier Organic Anion Transporter Family Member 1B1*) gene, encoding a hepatic transporter responsible for statin uptake, increase susceptibility to muscle-related adverse effects, particularly with simvastatin [98]. Identifying carriers of risk alleles allows clinicians to adjust doses, monitor more closely, or switch to alternative statins, thereby maintaining efficacy while minimizing toxicity. Incorporating this information enhances patient safety and adherence by mitigating drug-related complications [99].

Communally, these examples highlight the transformative potential of NGS-guided pharmacogenomics. By integrating genetic information into prescribing decisions, clinicians can personalize therapy, optimize drug response, and reduce adverse events, advancing the broader goals of precision medicine (Table 4).

Beyond pharmacogenomics, advances in genomic medicine are paving the way for novel precision therapeutic strategies in cardiovascular disease. Gene-based interventions, including antisense oligonucleotides, RNA interference technologies, and emerging CRISPR/Cas9-mediated genome editing approaches, hold potential for directly targeting disease-causing variants [100,101]. In parallel, therapies guided by genetic findings, such as PCSK9 inhibitors for familial hypercholesterolemia or genotype-specific management in inherited cardiomyopathies, demonstrate the expanding role of genomics in therapeutic decision-making [102]. Although many of these approaches remain in experimental or early clinical stages, they underscore a shift toward mechanism-based, individualized treatment strategies in cardiovascular care.

## 7. Challenges

A significant challenge in current NGS is the frequent identification of genetic variants whose clinical significance remains uncertain. Determining their relevance often requires comprehensive population-level data, family-based segregation studies, and functional assays. Another concern is the detection of incidental findings, pathogenic variants unrelated to the initial testing indication, which raises complex ethical questions regarding disclosure and informed consent [103].

Technical and interpretive limitations further complicate NGS implementation. Certain genomic regions, such as those with a high GC content, repetitive sequences, or highly homologous pseudogenes, can be difficult to sequence reliably. Additionally, the identification of large structural variants and copy number variations (CNVs) often requires specialized bioinformatics workflows, with WGS typically outperforming WES or targeted gene panels in detecting such complex alterations. These factors underscore the importance of rigorous quality control, confirmatory testing when necessary, and transparent reporting of assay limitations [104].

Integrating genomic data into clinical practice necessitates a multidisciplinary approach, involving cardiologists, clinical geneticists, genetic counselors, and bioinformaticians [105]. Despite technological advances, several barriers persist, including disparities in testing availability, inconsistent insurance coverage, and a knowledge gap among non-genetics healthcare providers regarding the interpretation and communication of results. Addressing these obstacles is critical to fully leverage NGS for personalized cardiovascular care, ensuring that genomic discoveries translate into meaningful clinical benefits while minimizing uncertainty and potential harm for patients [106].

## 8. Future Directions

Genomic technologies are advancing at an unprecedented pace, offering the potential for profound insights into cardiovascular biology and disease mechanisms. New approaches are expanding the clinical utility of genomics in cardiology. Long-read sequencing enables the accurate characterization of structural variants, complex genomic rearrangements, and epigenetic modifications that are often missed by conventional short-read techniques. These capabilities are particularly valuable in cardiovascular disease, where rare copy number variations, tandem repeats, and non-coding regulatory variants can contribute to conditions such as cardiomyopathies, congenital heart defects, and arrhythmias [107]. For instance, long-read approaches have identified structural variants in *TTN,* which is associated with cardiomyopathies, improving both diagnostic yield and genotype–phenotype correlations [108]. Integration of long-read sequencing with traditional genomic and clinical data holds promise for enhancing precision diagnostics, risk stratification, and targeted therapeutic strategies in cardiovascular medicine.

Single-cell sequencing now allows the investigation of how individual cardiac cell types respond to stress or pathology, while multi-omics integration, combining genomic, transcriptomic, and proteomic data, provides a comprehensive, systems-level understanding of cardiovascular function and dysfunction [109]. In CAD, single-cell studies have revealed heterogeneity among endothelial cells, smooth muscle cells, and immune infiltrates within atherosclerotic plaques, highlighting specific pro-inflammatory and pro-fibrotic subsets that may drive plaque progression and instability [110]. Integrating single-cell data with genomic, epigenomic, and clinical information promises to enhance precision diagnostics, identify novel therapeutic targets, and enable patient-specific risk stratification in cardiovascular medicine.

Artificial intelligence (AI) is increasingly applied to genomic data, automating variant prioritization and pattern recognition to assist clinicians in interpreting complex datasets. In parallel, population-scale genomic screening programs, such as proactive NGS-based testing for actionable conditions like familial hypercholesterolemia in biobanks or at birth, offer the opportunity to identify high-risk individuals before disease manifests. Early identification can facilitate timely interventions, potentially preventing premature cardiovascular events on a large scale [111].

**Figure 6:**
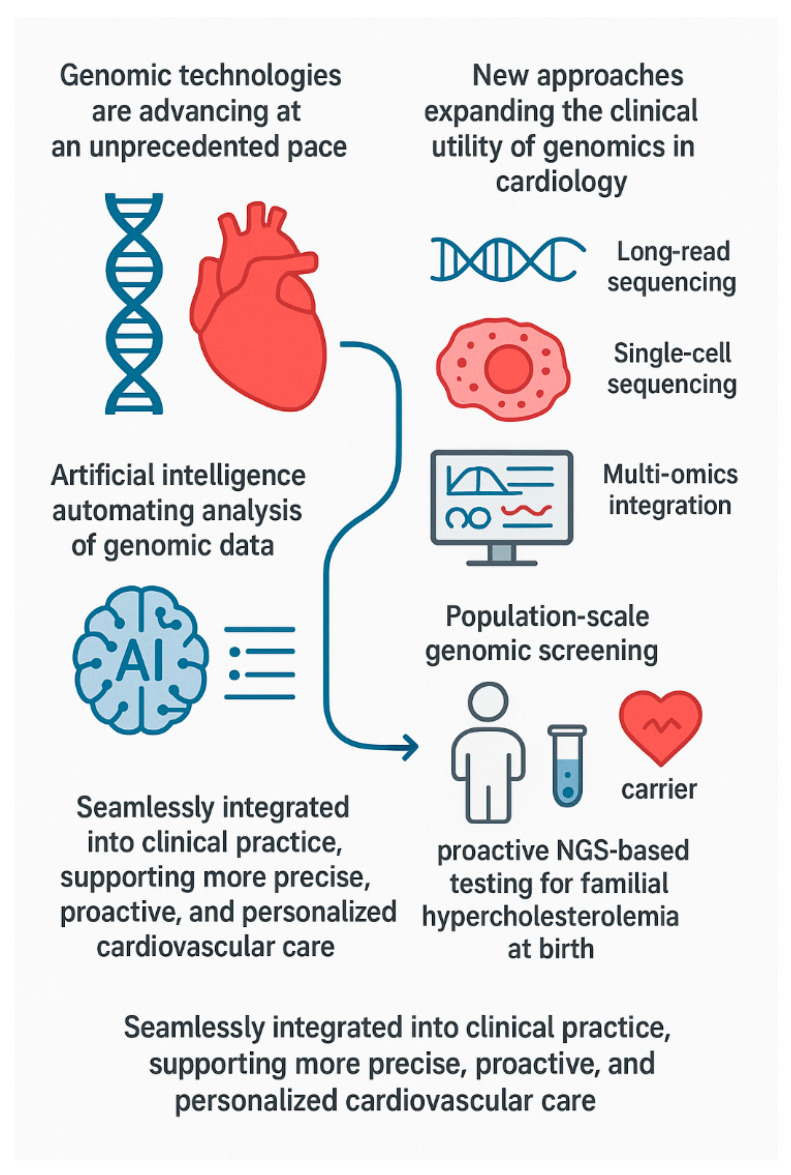
The evolving landscape of genomic technologies in cardiovascular medicine. This conceptual diagram illustrates the key technological pillars driving the evolution of cardiovascular genomics and their integration toward precise, proactive, and personalized patient care.

Together, these innovations signal a future in which genomic insights are seamlessly integrated into clinical practice, supporting more precise, proactive, and personalized cardiovascular care (Figure 6).

## 9. Conclusions

Modern cardiovascular genetics increasingly depends on NGS to unravel both rare inherited syndromes and the cumulative influence of multiple genetic variants. While challenges in variant interpretation and ethical considerations remain, innovations such as long-read sequencing, single-cell genomics, integrative multi-omics, and artificial intelligence-assisted analyses are progressively enhancing the accuracy and clinical utility of genomic data. As these approaches become embedded in routine cardiology practice, they hold the promise of transforming care from a predominantly reactive model to one that is preventive, personalized, and tailored to the individual patient’s genomic profile.

## Figures and Tables

**Table 1:** Comparison of NGS approaches.

Feature	Targeted Gene Panels	Whole-Exome Sequencing (WES)	Whole-Genome Sequencing (WGS)
Genomic Coverage	Focused on 50–200 clinically relevant genes	Covers ~1–2% of the genome (coding exons only)	Comprehensive coverage (~100% of the genome, including exons, introns, and non-coding regions)
Diagnostic Yield	High for known genes	Moderate to high	Highest, enabling detection of novel and rare variants
Turnaround Time	Fast (~2–4 weeks)	Intermediate (~4–8 weeks)	Longest (~8–12+ weeks)
Cost	Lowest	Moderate	Highest
Data Storage Requirements	Minimal (gigabytes)	Moderate (tens of gigabytes)	Extensive (hundreds of gigabytes)
Variant Interpretation Complexity	Relatively straightforward	Moderate complexity	Most complex due to non-coding regions, structural variants, and variant density

**Table 2:** Genetic and clinical characteristics of major inherited cardiomyopathies.

Cardiomyopathy	Key Genes	Typical Variant Types	Approx. Diagnostic Yield	Inheritance Pattern	Typical Age of Manifestation	Representative Clinical and Genetic Features
Hypertrophic Cardiomyopathy (HCM)	*MYBPC3, MYH7, TNNT2, TNNI3, TPM1*	*MYBPC3* truncating variants; *MYH7* and others usually missense	~30–60% in adults with confirmed phenotype	Autosomal dominant	Usually adulthood; *MYH7*-related forms may appear earlier	Pathogenic variants determine arrhythmic risk and heart failure progression. Identification of causative variants enables genotype-guided management and targeted family screening.
Dilated Cardiomyopathy (DCM)	*TTN, LMNA, DSP, FLNC, RBM20, TNNT2, MYH7*	Primarily truncating (*TTN*, *LMNA*); occasional missense (*LMNA*, others)	~15–25% among familial cases (highest for *TTN*)	Predominantly autosomal dominant	Typically, adulthood; *LMNA* variants can present earlier	*LMNA*-related disease carries elevated risk of malignant arrhythmias and may warrant prophylactic ICD placement. Expression is variable; family-based genetic evaluation is recommended.
Arrhythmogenic Right Ventricular Cardiomyopathy (ARVC)	*PKP2, DSP, DSG2, DSC2, JUP*	Mainly truncating and missense variants	50–60% in individuals with a positive genotype, especially *PKP2*	Autosomal dominant with incomplete penetrance	Usually, adolescence to early adulthood	High-intensity exercise accelerates progression and arrhythmic events. Genetic confirmation informs risk counseling, activity restriction, and cascade testing in relatives.

**Table 3:** Genetic and clinical overview of inherited arrhythmia syndromes.

Syndrome	Key Genes	Inheritance Pattern	Approximate Genetic Detection Rate	Common Triggers or Clinical Features	Clinical Utility of Genetic Testing
Long QT Syndrome (LQTS)	*KCNQ1 (LQT1), KCNH2 (LQT2), SCN5A (LQT3)*	Autosomal dominant (most forms, including LQT1–3); autosomal recessive in rare Jervell and Lange-Nielsen syndrome	>75% in patients with a confirmed clinical diagnosis	LQT1: arrhythmias during physical activity (swimming); LQT2: triggered by emotional stress or sudden noise; LQT3: more likely at rest or during sleep	Supports genotype-informed risk stratification, guides tailored therapy (β-blockers or sodium channel blockers), and enables targeted family screening
Brugada Syndrome (BrS)	*SCN5A* (most commonly implicated)	Autosomal dominant with variable penetrance	~20%	Arrhythmic events triggered by fever, certain medications, electrolyte imbalance	Confirms diagnosis in selected cases; helps identify at-risk relatives, though a negative result does not exclude the condition
Catecholaminergic Polymorphic Ventricular Tachycardia (CPVT)	*RYR2* (dominant), *CASQ2* (recessive)	*RYR2*: Autosomal dominant; *CASQ2*: autosomal recessive	~60% in clinically affected individuals	Ventricular arrhythmias during exercise or emotional stress; resting ECG usually normal	Critical for definitive diagnosis, guides preventive measures (β-blockers, activity restriction), informs cascade family screening

**Table 4:** Pharmacogenetic implications for selected cardiovascular drugs.

Drug	Relevant Gene(s)	Genetic Impact	Recommended Clinical Action
Warfarin	*VKORC1*, *CYP2C9*	Genetic variants alter dose requirement and sensitivity	Employ genotype-informed dosing algorithms prior to therapy
Clopidogrel	*CYP2C19*	Poor-metabolizer phenotypes reduce active metabolite generation and efficacy	For poor metabolizers, switch to alternative P2Y_12_ inhibitors (e.g., prasugrel or ticagrelor)
Simvastatin	*SLCO1B1*	Reduced transporter function increases risk of myopathy	Avoid high-dose simvastatin in at-risk genotypes; consider an alternative statin
Beta-blockers	*ADRB1*	Variant alleles may modify receptor response	Consider genotype when evaluating response and dose, as evidence evolves

## Data Availability

The original contributions presented in this study are included in the article. Further inquiries can be directed to the corresponding author.

## Abbreviations

The following abbreviations are used in this manuscript:

**Abbreviation**	**Full Term**
ACM	Arrhythmogenic Cardiomyopathy
AD	Autosomal Dominant
AF	Atrial Fibrillation
AR	Autosomal Recessive
BP	Blood Pressure
CAD	Coronary Artery Disease
CNV	Copy Number Variation
CMP	Cardiomyopathy
CV	Cardiovascular
DCM	Dilated Cardiomyopathy
DNA	Deoxyribonucleic Acid
ECG	Electrocardiogram
EF	Ejection Fraction
ExAC	Exome Aggregation Consortium
GWAS	Genome-Wide Association Study
HCM	Hypertrophic Cardiomyopathy
HTN	Hypertension
INDEL	Insertion–Deletion Mutation
LDL	Low-Density Lipoprotein
LQTS	Long QT Syndrome
LV	Left Ventricle
MAF	Minor Allele Frequency
MI	Myocardial Infarction
mRNA	Messenger Ribonucleic Acid
NGS	Next-Generation Sequencing
PCR	Polymerase Chain Reaction
RNA	Ribonucleic Acid
SNP	Single-Nucleotide Polymorphism
SV	Structural Variant
VAF	Variant Allele Frequency
VF	Ventricular Fibrillation
VT	Ventricular Tachycardia
VUS	Variants of uncertain significance
WES	Whole-Exome Sequencing
WGS	Whole-Genome Sequencing
